# Promoting healthy foods among urban school children in Bangladesh: a qualitative inquiry of the challenges and opportunities

**DOI:** 10.1186/s12889-021-11085-0

**Published:** 2021-06-01

**Authors:** A. M. Rumayan Hasan, George Smith, Md Harunor Rashid, Mohammad Abdus Selim, Sabrina Rasheed

**Affiliations:** 1 Universal Health Coverage, Health Systems and Population Studies Division (HSPSD), International Centre for Diarrhoeal Disease Research, Bangladesh, 68 Shaheed Tajuddin Ahmed Sarani, Mohakhali, Dhaka, 1212 Bangladesh; 2 Fragments Magazine, Dhaka, Bangladesh

## Abstract

**Background:**

In Bangladesh overweight and obesity among urban school children are on the rise. Urban school children tend to consume foods dense in calories and few fruits and vegetables which is associated with overweight and obesity. The current study explored the barriers and opportunities for promoting healthy diets among school children from the perspective of teachers and parents in Dhaka, Bangladesh.

**Methods:**

We conducted 14 key informant interviews with teachers and principals, six focus group discussions with 31 mothers of school children (5 to 15 year old) and 14 structured observations of the school food environment. Inductive thematic analysis was performed manually.

**Results:**

Schools were important for development of food preferences of children, however, most school cafeterias provided foods based on profit rather than health considerations. A shift in food culture resulted in making eating out acquire many meanings beyond convenience. Mothers, especially those who were employed, struggled to prepare healthy foods due to time pressure. Mothers were generally concerned about chemicals added to raw foods in markets which led to limited fruit and vegetable consumption.

**Conclusions:**

There were many challenges to promoting healthy foods to school children within and outside the school. It is important to formulate policies and guidance to create a supportive environment for healthy foods in and in the proximity of schools. It is also important to educate consumers about identifying and choosing healthy foods. Laws related to food safety should be adequately implemented to boost the population’s confidence in safety of available healthy foods in the food system.

**Supplementary Information:**

The online version contains supplementary material available at 10.1186/s12889-021-11085-0.

## Introduction

The rapid global increases of childhood overweight and obesity herald an urgent need to understand how to address childhood risk for adiposity [1–3]. Bangladesh is experiencing a double burden of malnutrition with high rates of under nutrition and rising rates of overweight and obesity (7.6 and 9.5%) among children and adolescents especially in urban areas [4, 5]. Developing healthy eating habits during childhood and adolescence is important as these stages of life come with increased demand for nutrients [6, 7], and eating habits developed during this age is likely to influence life-long healthy food consumption behavior [8, 9]. Healthy eating habits have been associated with increased educational achievements, cognition [10, 11] and better health [12]. Despite the importance of healthy diets, researchers have reported that the diets of children and adolescents are rich in fat, salt and sugar, and lacking in vegetables, fruits, whole grain and calcium-rich foods [13–16]. Among urban Bangladeshi school children researchers found high consumption of fast foods and sugary drinks, and low consumption of fruits, vegetables and animal source food [17, 18]. However, little is known about the contextual drivers of these food choices.

Social, cultural, and environmental factors have been shown to influence eating habits of school children and adolescents [19–22]. Researchers have suggested that characteristics of the school environment such as the availability of healthy foods, school nutrition and wellness policies and provision of nutrition education affect children’s food consumption behavior [23–25]. The social environment of the school and peer influence can also affect food choices [20, 26, 27]. At the household-level, types of food available, time available for food procurement and preparation, maternal education and employment, parental food preference and consumption of foods prepared outside home significantly affect children’s food habits [28–30]. Several recent studies have demonstrated that mass media advertisements increase the desire for unhealthy foods among children and adolescents [20, 31]. In Bangladesh school lunch policies and program have been developed as a part of social safety net to address problems of household food insecurity and primary school drop out [32, 33]. Currently in Bangladesh, school feeding program are available in 35 of 64 districts covering 1500 rural primary schools and approximately 3 million students mainly focusing on district with higher levels of poverty [34]. However, to date, there are no guidelines available on provision of foods in school cafeteria and no efforts have been made to overtly link the existing health education module of the curriculum to provision of food within schools.

Given the gaps in knowledge as well as policies and programs, it is important to examine the contextual drivers of food habits among school-going children and adolescents. With this paper we attempt to understand the barriers to and opportunities for promoting healthy diets for school children in households and schools, from the perspective of teachers and parents in urban Bangladesh.

## Materials and methods

### Study design and study site

The current study was part of a larger cross-sectional study designed to assess the risk factors of overweight and obesity among school children. For our current analysis we focused on qualitative data related to healthy food consumption. The study was conducted in 14 randomly selected schools (Table 1) in two purposively chosen administrative areas of Dhaka city from January–June 2018. After mapping all schools in the selected areas and we stratified them into low (USD 4–24), medium (USD 25–70) and high tuition (USD 71–276) school based on the monthly school fees. The assumption was that the tuition rates would represent the socioeconomic status of the students and their families. To ensure an equal male-female ratio, if a girls’ school chosen, we selected additional boys’ school from the same area. Finally we selected 14 schools [35]. .

**Table 1:** Sampling of schools by study area, medium of instruction, types of school and tuition fees [35]

Characteristics	High-tuition schools	Medium-tuition schools	Low-tuition schools
Area			
Dhaka North	2	2	3
Dhaka South	2	2	3
Medium of instruction			
English only	3	0	0
Bengali/English	1	0	0
Bengali only	0	4	6
Types of school			
Co-education	4	4	4
Single sex	0	0	2

### Participants and procedure

We conducted 14 key informant interviews (KIIs) with school teachers and principals, six focus group discussions (FGDs) with mothers and structured observations of the food environment (based on a 14-item checklist) on the school premises. First, we provided formal letters to authorities from all selected schools explaining the objectives and process of the study, and inviting each school to take part. We asked all school principals to participate as key informants as they were responsible for the overall administration of the school. Based on principals’ recommendations, teachers who were responsible for delivering nutrition education (as part of the curriculum) were interviewed. Initially we planned to recruit both fathers and mothers of school children for the FGDs, but only mothers were available in waiting areas (as they waited for their children) adjacent to the schools. Therefore, we only included mothers in the FGDs. Purposive sampling was used to recruit the participants for both KIIs and FGDs.

### Data collection

Four researchers (2 males, 2 females) with Masters in either Anthropology or Social Science and extensive experience in qualitative data collection, conducted KIIs, FGDs and observations. KIIs were arranged at convenient times and places for the teachers (usually their office during school hours). FGDs were held in waiting areas adjacent to the schools. We conducted structured observations of all available school cafeterias.

Open-ended interview guides for KIIs and FGDs were developed based on a literature review of food habits of school children. The guides were pre-tested in a school that was not selected for the study. The guides for both the KIIs and FGDs were revised based on pre-testing. The KII guide explored: a) Perceptions about healthy foods and concerns about food adulteration; b) foods available at school cafeterias; c) food preferences of students; and d) barriers to promoting healthy foods in cafeterias. From parents’ perspectives the FGD guide explored: a) understating of and attitude towards healthy foods; b) concerns about food safety; c) children’s food preferences; and d) perceived barriers and facilitators to providing healthy food both within and outside of school. A checklist was used for conducting structured observations. The list covered information about the cafeteria facilities, available foods and food safety measures. The checklist was prepared based on a literature review on food environments and the checklist was pre-tested to ensure its applicability in our context. Extensive field notes were taken during the interviews and FGDs. The KIIs lasted 40–50 min and the FGDs 60–90 min. The interviews and discussions were audio-recorded and transcribed in Bengali verbatim. Two investigators (RH and AS) checked the transcribed data against the original recordings for accuracy and completeness, and any differences were reconciled. Data collection ended when data saturation was reached.

#### Ethics statement

The Ethical Review Committee of icddr,b approved the study. All participants provided written informed consent before the interview or FGD and permission was sought for audio-recording. The purpose of the study, anticipated duration of the KII or FGD, confidentiality and participants’ right to withdraw from the study were clearly explained in the consent form. We offered time to participants to go through the consent form and ask questions prior to the interview or FGD. Each participant was assigned an ID number to maintain anonymity during data analysis.

### Data analysis

**Fig. 1:**
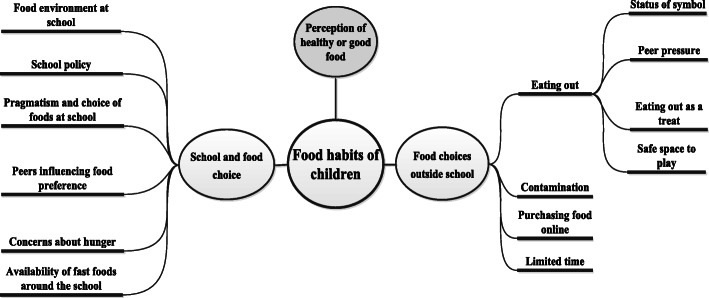
Themes of qualitative exploration

We used inductive thematic analysis procedures manually [36]. These analytical procedures were undertaken in 6 stages [37]: a) becoming familiar with the transcribed data through repeated readings; b) generating initial codes and gathering data under each code; c) identifying themes and sub-themes and extracting data into themes and sub-themes related to environmental and social barriers to healthy food; d) reviewing themes and formatting a thematic matrix for further analysis; e) defining and naming themes; and f) write up. Two investigators (RH and AS) independently reviewed all transcripts and applied codes to the data. They cross-checked each other’s coded transcripts and discussed and resolved any discrepancies in coding by consensus. For triangulation, we compared and contrasted findings from the KIIs, FGDs and observations. The team regularly met to understand the key issues and to discuss interpretations of the data. Based on the analysis the themes we arrived at are provided in Fig. 1.

## Results

### Characteristics of respondents

The FGD respondents mostly were stay-at- home mothers and were 30–39 years of age, and had 11–15 years of education. Most of the key informants were male and were 40–49 years of age, and had more than 11 years of education (Table 2).

**Table 2:** Respondent profile [35]

Characteristics		
	Focus group discussion (*n* = 31)	Key informant interview (*n* = 14)
***Age group***		
20–29	8	0
30–39	16	0
40–49	7	10
50–59	0	4
***Gender***		
Male	0	9
Female	31	5
***Profession***		
Stay at home mom	24	0
Service	7	0
Teacher	0	14
***Years of schooling***		
6–10	11	0
11–15	20	14

#### Mothers’ perceptions of healthy/good foods

When asked what they consider to be “good” food mothers mentioned foods normally cooked at home and fruits and vegetables. Most mothers thought that foods prepared at home were safer than those prepared outside the home. Many mothers mentioned rich foods such as biriyani and meat and *polau* (pilaf) as good (good to taste or prized) food. Some mothers talked about balanced diets and the need for a variety of foods to arrive at a balanced diet. A few mothers of children studying in medium- and low-tuition schools thought that fast foods such as deep-fried chicken, *shawarmas* (meat and vegetables wrapped in flatbread) and burgers were good food for their children which lead these mothers to prepare these foods at home when they could not afford to buy them. Most mothers, however, thought that fast foods were popular among children but unhealthy. This perception was also held by teachers, who were very concerned about student nutrition. As one principal mentioned:

I noticed that in the past our students liked to eat foods prepared at home. Now children do not like these foods. Nowadays children like and want to eat “better food” such as meat, fast food, sugary drinks, burger, chips and other fried and sweet foods available in the market. They eat these foods from school, outside school and their parents make these foods at home as well. These foods are harmful for health and if eaten regularly can lead to obesity. (KII-3_principal, medium-tuition school)

Some detrimental effects of fast foods mentioned by mothers were stomach problems (diarrhea, dysentery, typhoid) and digestive problems (heart burn, peptic ulcer). Very few mothers associated weight gain with consumption of fast foods; all the teachers mentioned this link, however no matter how a mother defined good food, it was clear that providing good food to their children was a symbol of love.

*My son is the only male in my entire family. He was born after 3 girls. We love him a lot so we keep the best food for him … mainly meats. When we take him to relatives’ house(s) they also try to offer meat-based food (chicken or beef). If meat was not cooked they cook the meat and then the meal is served … never without meat. They do this because they love him. (FGD-2_mother, medium-tuition school)*

In the next section of the paper we will discuss the existing food environment in and outside schools that shapes school children’s eating pattern.

#### Schools and food choice

##### The food environment at school

During our observation of school premises, we found that 10 out of 14 schools had a cafeteria from which children could buy food and drinks during breaks. Some cafeterias had seating arrangements. Based on our observation only at high-tuition schools were all the foods for sale kept in covered displays; in other schools covering foods was sporadic (Table 3). In most schools, food service was contracted out to vendors who prepared the food outside the school premises. Only the high-tuition school cafeterias had space for food preparation.

**Table 3:** Facilities available in the cafeteria

Facilities available	High tuition school (***n*** = 4)	Medium tuition school (***n*** = 4)	Low tuition school (***n*** = 6)
Cafeteria available	3	3	4
Food prepared on school premises	3	0	0
Foods prepared outside school premises	0	3	4
Foods were covered	3	2	1

Most school cafeterias sold local snacks, fast foods and sugary, carbonated drinks (Table 4). The foods sold were generally calorie dense and none of the schools provided the option of fresh fruits. Among schools that participated in our study only one (high-tuition) had a policy against selling carbonated drinks.

**Table 4:** Types of foods available in school cafeterias

Types of foods available	High-tuition schools (***n*** = 3)	Medium-tuition schools (***n*** = 3)	Low-tuition schools (***n*** = 4)
Local snacks^*^	2	3	4
Fast foods^#^	3	2	1
Meal packets^@^	2	0	0
Sugary drinks	2	2	2

^*^ *Singara (*Potato deep fried in a dough*), somusa (*similar to singara*), peaju (*lentil fritters*), chotpoti (*boiled chick pea in spiced tamarind sauce*), chola (brown gram fried in spices),* Jhal muri *(puffed rice mixed with oil and spices)*

^#^Fried chicken, French fries, burgers, sandwiches, rolls, patties, *shawarma* (wrap)

^@^Tehari, biryani, khichuri

##### School policy

None of the schools had specific policies about providing healthy foods and drinks for their students. We found that carbonated drinks (all sugar-sweetened) were sold in the majority of school cafeterias. Only one high-tuition school banned carbonated drinks for concerns about rising rates of overweight and obesity among students. The principal explained:

*We don’t allow (carbonated drinks) in our school. You know that these drinks are the main reason why kids are gaining weight. These are not available in our cafeteria and we do not allow them in our premises. This rule was made only out of health concerns.* (KII-7_principal, high-tuition school)

##### Pragmatism and choice of foods at schools

During our discussion with school principals we inquired about how decisions were made regarding the types of foods that would be available in the cafeterias. All the principals mentioned that student preference was the main driver for the decisions. In some cases attempts had been made to provide healthier food options, but if students had not bought them, the schools had stopped offering. As a principal at a high-tuition school said:

*We know that students prefer fast food and junk food and so we sell these in the canteen. Once we kept some fruits for sale but they did not sell. Even if (healthier foods) are available, (students) are interested to buy fried chicken, French fries or singara. Thus, we stopped offering. Our students cannot seem to accept fruits in the school canteen.* (KII-7_ principal, high-tuition school)

Profit was an important factor for deciding on the types of foods to be sold in school cafeterias. Most schools leased their cafeterias out to an external company or person and the lease agreements did not have clauses about quality and types of foods that should be provided. The company or person who got the contract was allowed to run the cafeteria and set the menu as they saw fit. So once the cafeteria was leased out, school authorities had little control over the foods offered.

*We always tell (the food provider) to make sure good quality food is provided … We tell them to reduce fried food and offer nutritious foods. We can’t make them listen. They have the lease to run (the cafeteria) … They have to make profit. They will have to provide foods that children are interested to buy … We have to see their perspectives too.* (KII-3_principal, medium-tuition school)

While school teachers spoke about the importance of student preferences for foods, mothers talked about students being compelled to purchase whatever foods the cafeterias offered. Mothers complained that the availability of unhealthy snacks at school stopped children from eating foods they sent from home.

*They offer mostly fast food … foods that are fried such as singara, somucha, sandwich, burger and hotdog. Children get fast food from the canteen daily. If these were not available (children) would have to eat foods that are provided from home.* (FGD-1_mother, high-tuition school)

##### Peer influence on food preferences

Mothers spoke about the strong influence peers had on children’s food preferences. Mothers noticed that after starting school food habits changed and their children often asked for foods that their friends ate at school. Although a few mothers initially resisted their children’s demand for foods they thought were not healthy, they usually gave in to the demands after a few days. Some mothers felt that their children would feel deprived if the preferred foods were not given.

*My son said, ‘My friend brought chicken ball. Please give me chicken ball too.’ I resisted his demand for 2 days and give in on the third day. When my son sees other eat this he will feel deprived.* [sic] (FGD-1_mother, high-tuition school)

Teachers also observed that the types of foods brought from home have changed based on children’s preferences.

*The students do not want to eat roti (flatbread), vegetables or curry at school. If parents provide these foods, he/she refuses to eat it. They have this concept that those foods cannot be eaten at school. They like to eat fried chicken (during recess) instead* (KII-5_phys ed teacher, medium-tuition school)

A few mothers were concerned about the teasing and ridicule their children would face if they did not conform to the habits of their peers.

*My son does not want to bring food (from home). He thinks that I will give him bread, vegetables and fried eggs to eat. He feels self-conscious about taking these foods to school. When he opens the box, his friends laugh at him.* (FGD-6_mother, low-tuition school)

##### Concerns about hunger

Mothers’ concerns about their children being hungry also resulted in them providing junk foods. Most mothers irrespective of the type of school their children attended told us that their children often skip breakfast, which compounded mothers’ worries about their children not eating during recess. The teachers also observed that many children came to school without eating breakfast. If mothers did not provide foods that their children liked, they worried that their children would not eat. Mothers believed that the lack of food for a long period hurt children’s ability to learn and to stay healthy. These concerns led to the provision of foods that children liked or allowing children to purchase junk foods from school cafeterias.

*If I give him homemade food, he does not eat it during recess. The (lunch) box comes home full … If I buy him fried chicken or chicken shawarma (from the shops) or give him money to buy (food), the food is never brought back (home). Sometimes he demands more money than he needs so that he can buy chicken fry in addition to the sandwich. I think that at least his stomach is full … I am compelled (to buy fast food) … He does not eat (food prepared at home).* (FGD-1_mother, high-tuition school)

The teachers observed that when children skipped breakfast they tended to eat more fast food or junk food during recess. A Headmaster explained:

*We noticed that most children come to school without eating breakfast. They do not get time to eat (after they come to school). That means that until 10 or 11 o’clock they remain hungry. So when they eat (during recess) they eat a lot and the food is usually junk food or fast food.* (KII-7_principal, high-tuition school)

##### Availability of fast foods around the school

The availability of fast foods near the schools influenced children’s access to them. Mothers spoke about children demanding that their parents purchase foods and soft drinks on the way back from school. The foods available on the streets outside schools are of low quality but many mothers give in to the demands. One mother at a high-tuition school described the situation in the following way:

*Every day (my daughter) comes out of (school) and insists on buying food (from the street). There are some fast food shops just outside the school and (she) demands that I buy the fast foods and cold drink. If I refuse she cries and throws a tantrum … It is embarrassing. I am forced … It is a big problem. I live very close to the school and she can come home to eat lunch but everyday she insists on eating these foods.* (FGD-4_mother, high-tuition school)

#### Food choices outside school hours

Beyond the food environment in and near schools, use of ready to use convenience foods, eating out and use of online apps to buy foods has become an important part of the family food ecosystem. While ready to eat and easy to prepare foods at breakfast are driven by school and work schedules, having family meals outside the home has many meanings beyond convenience.

##### Limited time

Lack of time to prepare foods for children was a common topic of discussion among mothers. In the mornings mothers struggled to prepare breakfast for the family and school snacks for their children. And mothers who worked outside the home struggled to get ready for work, themselves, in addition to helping their families start the day. The limited time available and lack of help meant that mothers used store bought, ready meals that can be prepared fast, such as sausages, nuggets and breads as opposed to more traditional breakfasts. Limited time in the morning also forced mothers to allow children to buy foods from school cafeterias.

*I wake up at 5:30am and make breakfast for everyone and tiffin (snacks) for children’s school. I have to take (my daughter) to school so I have to get out of the house by 7am. I have to do a lot of work (within this short time) you understand? Making roti (flatbread) and shobji (vegetables) take time and effort. There are foods (such as) chicken nugget, sausage and ball … they are available frozen at the store … I keep them in the fridge and easily fry them or breakfast. I have to say these foods have made our life easy and children like them.* [sic] (FGD-3_mother, medium-tuition school)

##### Contamination

While acknowledging the importance of fruits, vegetables and fresh home-made foods, all mothers expressed concern about adulterants such as formalin added to raw foods in the markets. Mothers thought chemicals added to foods post-harvest were harmful for health and worried about giving such adulterated foods to their children. The concerns related to food adulteration also encouraged a few mothers to refrain from buying fruits and to favor fast foods.

*We want to give (children) nutritious food but we can’t because (the food) has formalin. Formalin causes harm to health. Nowadays there is formalin in fruits and vegetables … We are very scared … Chicken is better. So we depend on fast food.* (FGD-3_mothers, medium-tuition school)

Interestingly none of the mothers mentioned health risks related to environmental contaminants or chemicals and pesticides used prior to harvest on fruits, vegetables and feed for livestock.

##### Purchasing food online

From the mothers with children in high-tuition schools we found that smart phone apps for ordering food online has made it easy for children to order foods from local restaurants and fast food outlets. The ease of ordering and delivery has increased access to and consumption of fast foods. Parents or children no longer have to go out to get the foods they want. As an administrator from a high-tuition school explained:

*There is a new trend (in eating food from outside home) … online order. Children can now order themselves. Many (children) go to bed after eating pizza. This was not possible (in the past) … Eating out frequently … you had to go to the restaurants. Now you don’t have to. Food will arrive (at home) within half-an-hour of order. Children ask and parents allow … This is increasing the consumption of fast foods.* (KII-7_ principal, high-tuition school)

#### Eating out

##### Status symbol

For mothers, particularly many with children in middle-tuition schools and a few with children in low-tuition schools, dining out was a symbol of social status. Mothers equated going to restaurants or fast food outlets with advertising the family’s financial wellbeing. So people went to restaurant to keep up appearances.

*Sometimes we eat (out) to maintain social status. I mean if we don’t eat out it feels (as if) we do not belong to a better class … It will feel as if we are not well off (financially) … so we visit restaurants*. (FGD-3_mother, medium-tuition school)

Interestingly, none of the mothers with children in high-tuition school mentioned this link between eating out and social status.

##### Peer pressure

Some mothers talked about peer pressure from their children’s friends leading to requests to go to specific food outlets to try different foods. Children often shared their experience of eating out in terms of specific foods and restaurant ambiance with their friends. The communication between friends inspires others to visit recommended restaurants or to eat recommended foods.

*My daughter tells me “my friends go (to restaurants), they eat fast foods. I want to go too”. I know that children talk to each other (about the experience of eating out). They like to talk (about eating out).* (FGD-2_mother, medium-tuition school)

However, it was interesting to note that mothers with children in low tuition-fee schools did not mention peer pressure from their children’s friends.

##### Eating out - a treat

As children were very interested in going out to eat, many mothers used eating at fast food outlets as a reward or treat to encourage or motivate their children to do tasks. One mother with children in a medium tuition-fee school said “*I often tell my son that if he finishes reading a certain book chapter, I would take him to his favorite restaurant. These rewards work well.”* (FGD-2_mother, medium-tuition school).

Mothers also talked about eating out to celebrate occasions such as birthdays demonstrating that eating out has become an integral part of family recreation.

##### Safe play space

Some fast food places have play areas for children. Children want to frequent these places to play and interact with other children. Mothers felt that they are good and safe places to take their children to play. However, the use of play areas was only allowed if one bought food. Sometimes even when mothers disliked the food, they brought their children to the fast food places to use the play area and to eat.

*We have to take my child to restaurants every week. They (restaurants) have play areas … My child likes to play … He can play with other children. You cannot access the play area just like that, you have to eat. The options are fast food. My child eats these.* (FGD 4_mother, high-tuition school)

Mothers with children in low-tuition schools did not mention using restaurant play areas.

## Discussion

In this study, mothers and teachers described social and environmental challenges in promoting healthy foods to children. Social and environmental barriers to promoting healthy foods among school children have been researched in various parts of the world but to our knowledge our study was the first to focus on the pathways through which different parts of home and school environments hindered parent’s ability to provide healthy foods to school children in urban Bangladesh. The insights from this study can help to formulate policies that support healthy food environments and contribute to a healthier Bangladeshi population in the future (Table 5). Our study has some limitations that need to be considered when interpreting the findings. First, we did not seek the experiences of children related to their food habits which could have enriched our findings. We did not include the perspective of owners of school cafeterias as they were not interested in being interviewed. Finally, we were unable to reach fathers as they were unavailable during school hours and, therefore, we missed their perspectives.

**Table 5:** Policy and program implications of the findings

Areas of interest	Constraints and barriers	Favorable factors	Opportunities for intervention
School food environment	- There are no guidelines for providing healthy food in schools - Cafeterias were contracted out and school authorities did not have any control over the foods and drinks offered	- Teachers are aware of the benefits of healthy foods - Parents were concerned about availability of healthy foods in cafeterias	- Policies and guidance to create a supportive environment for healthy foods in schools can be developed - Contracts with food vendors should include mandatory provision of healthy and safe foods as well as periodic monitoring.
Food adulteration	- Mothers were concerned about chemical contamination of foods -Perception of food adulteration led to limited fruit and vegetable consumption	-Mothers were aware of the need for healthy food. -Mothers considered fruits and vegetables as healthy foods.	- Adequate implementation of food safety laws is needed - Provision of both punitive and supportive measures should be made in the different laws to encourage the food industry to comply with food safety laws - Real strides in food safety must be made in order to legitimately increase community trust in the safety of food systems
Convenience foods and foods prepared outside home	-Mother struggled with lack of time to prepare healthy foods -Apps allow children and parents to purchase food online	-Parents are concerned about the health of children - Apps make purchasing food easy	-Consumers should be provided with easy ways of identifying healthier options among foods available in the different markets - The restaurant industry should be compelled to provide healthy options

In our study we found that mothers, especially those who were employed, struggled with having adequate time to prepare healthy foods for their children. This lack of time should be understood in the context of nuclear families [38], which reduces options for help; and mothers joining the workforce [39] has further diminished time. The ensuing time pressure has created a niche for packaged, pre-prepared foods and for fast foods. Home cooked, minimally processed foods being replaced by ultra-processed foods is problematic because processed foods often are high in sugar, fat and sodium [40, 41] and high consumption of such foods has been associated to overweight, obesity and NCDs [41, 42]. From studies of food systems researchers have reported that the powerful influence of advertisements led to the introduction of new foods into diets and the emergence of retail outlets which in turn increased access to the new foods [43], all of which added up to a shift in food culture [44].

Beyond the convenience of pre-prepared foods, we found that eating out has become normative as it has acquired new meanings beyond sustenance. With rising urbanization in developing countries [45] and the increase in women’s workforce participation [39], it is important to learn from already existing best practices to improve health through diets. These best practices incentivize the reduction of fats, sugars or sodium in targeted products. Such specific programs include the sliding scale health promotion levy in South Africa [46], mandatory calorie labels in restaurant chains [47] and marketing bans on foods that don’t meet quality standards [48]. In addition to ingredient specific food standards, Bangladesh can also consider making less processed alternatives affordable for a wide range of population through subsidies [49]. But if we are to promote less processed foods and freshly prepared foods, we must deal with the lack of time for food preparation in today’s world. Ways to address lack of time for mothers include enforcing maximum working hour mandates, legislating for and enforcing stable employment contracts, rethinking school schedules and ensuring that we creatively deal with the deeply ingrained cultural notion that food preparation is almost exclusively the responsibility of girls and women. Therefore it is important to make food studies mandatory for both boys and girls – inculcating life-long food skills [50].

We found that school played an important role in shaping food preferences, sometimes probably to the detriment of student health. Although curricula included lessons about healthy foods, cafeterias sold foods based on profit considerations rather than health. In many cases the school cafeterias were contracted out and school authorities lacked influence on cafeteria foods and drinks. Similar findings about school cafeterias serving energy dense and low quality foods to maximize profit have been reported from both developed and developing countries [51–53]. This is an unfortunately poor choice of priorities and a missed opportunity. With food and nutrition already part of an existing school curriculum, school cafeteria and school lunch programs can be used to practically implement the lessons learnt which would deepen the understanding of healthy foods. No matter the state of the resources available to them guidelines should be developed to help schools implement promotion of healthy diets in schools. For example, whether schools have their own cafeteria or allow outside vendors to sell food within their premises, the guideline should articulate a standard quality of food both in terms of health and hygiene; require mandatory training for caterers and food vendors; and provide simple indicators for periodic monitoring by teachers and students. Existing literature points to the direct positive influence school food environment policies such as, establishing school meal standards, making fruit and vegetables (preferable from local and safe sources) available, and restricting sweetened drinks and unhealthy snacks, on children’s diets. In our study mothers mentioned that their children started disliking home made traditional snacks and started demanding foods prepared outside home especially fast foods after attending school. The indirect effect of having a school environment that supports healthy foods could work through peers at school which plays an important role in shaping children’s food preference in and outside school [20, 54, 55].

Currently there are no policies in Bangladesh that exist to address the school environment holistically. In recent years the Bangladesh Ministry of Education has drafted a policy on school feeding programs for primary schools [32] which is part of the social safety program focusing on addressing household food insecurity and primary school drop out. While this policy is a step, all schools – both public and private regardless of resources available – need policies and the political commitment to rigorously enforce a common guidelines for healthy eating among school children if Bangladesh is to create a supportive environment for healthy foods across its educational institutions.

In our study mothers were concerned about contaminants in fresh foods. Adulteration of fruits with formalin was specifically mentioned, but there was also deeper general concern about chemicals being added to raw foods in markets. Similar concerns about adulterants were reported by researchers from other countries [56, 57]. While acknowledging the importance of fruits and vegetables for a healthy diet, mothers in our study perceived that chemicals added to fruits and vegetables were harmful which led to them limiting their children’s fruit and vegetable consumption. Similar findings about safety concerns leading to reduced fruit and vegetable purchases were reported for Myanmar as well [58]. In other studies researchers have found that more than half of the foods consumed in Dhaka city were either adulterated or contaminated with toxic compounds (DDTs, formalin, textile dyes) or harmful microorganisms [59–61], leaving Dhaka residents uncertain about the quality of the foods available. This situation and these findings underscore the importance of restoring the trust on food systems so that safe foods become the expected norm among the consumers. It is important therefore, that policies dealing with food safety consider incentives for safe production practices; make safer alternatives such as refrigeration (for both preservation and transportation) available for perishable foods and articulate effective measures for monitoring enforcement. Presently, there are many laws related to food safety across government of Bangladesh ministries, enforcement is poor [62]. It is urgent for the health of its population that Bangladesh adequately enforces food safety laws.

It is glaringly obvious from our study that diet – one of, if not *the* primary driver of physical health – is being nearly completely left to the vagaries of the marketplace where health is just a cost to be externalized. It is vital that multi-sectoral policies are developed and implemented with a focus on health, rather than allowing the ideology of profit to dictate health. Allowing market players to drive the society’s diet is short sighted and highly costly even in economic terms alone (out of pocket expenditure due to illness and reduced productivity), let alone in terms of physical and mental health and wellbeing of the population. In low resource setting such as Bangladesh, it is crucial that the school environment be utilized to foster healthy habits through development and implementation of guidelines that promote healthy eating and adequate exercise. Involving parents in promoting healthy food would help to educate households and help them to make good dietary decisions for their children. Investing in the school environment and wider food system so that they are supportive to safe and healthy foods is imperative and must receive adequate attention.

## Conclusions

There were important social and environmental barriers for promoting healthy foods to school children. Most school cafeterias provided foods based on profit considerations rather than health. This despite concerns from both parents and teachers, which shows that there is a need for policies, guidance and enforcement to create a supportive environment for healthy foods across educational institutions. Processed foods and foods prepared outside home have become an important part of the urban food culture and have meanings beyond convenience which point to the need for intervention to promote healthy foods. Mothers did not trust the safety of raw foods in markets, specifically fruits and vegetables. It is important therefore, to develop and implement strict regulations and guidelines to ensure safe foods. It is also important to implement existing laws related to food safety so as to legitimately boost people’s confidence in the integrity of the food system.

## Supplementary Information

**Additional file 1:.**

## Data Availability

The datasets generated and/or analyzed during the current study are not publicly available based on the data access policy of icddr,b but are available from the corresponding author based upon reasonable request.

## Abbreviations

FGD: Focus Group Discussion
KII: Key informant Interview
Icddr,b: International Centre for Diarhhoeal Disease Research, Bangladesh
DDT: Dichloro Diphenyl Trichloroethane
NCD: Non Communicable Disease
