# Supplementary material for: Promoting healthy foods among urban school children in Bangladesh: a qualitative inquiry of the challenges and opportunities
Source: BMC Public Health. 2021 Jun 1;21:1029. doi: 10.1186/s12889-021-11085-0 (PMC8168019; doi:10.1186/s12889-021-11085-0)
Supplement: Supplementary file 1 — Additional file 1:. [file 12889_2021_11085_MOESM1_ESM.docx]

**Guideline for FGD**

1. What do you think about healthy foods for your children? What is your opinion about food adulteration?
2. Do your children eat anything before going to school? Why (not)? What they eat during break at school? With who? Do they buy it at school or take it from home? Why? Probe
3. What types of food do your children prefer to eat (At school/outside home)? Why they prefer to eat such food?
4. What factors do you perceive influence your children's food choice at school or outside school?
5. What are the barriers do you face in providing healthy foods to your children? How can overcome such barrier?

**Guideline for Key Informant Interview (KII)**

1. What do you think about healthy foods for school children?
2. What is your opinion about food adulteration?
3. What are the food facilities available in your school? What goes into the decision about what food serve in the cafeteria? (if any).
4. What types of food do children prefer to eat (At school)? Why they prefer to eat such food?
5. What are the challenges that impede ensuring healthy food at school? How can such challenges be overcome?
6. What kind of policies do your schools have about school food environment? (if any).
